# Application of Phosphogypsum in Ultra-High-Performance Concrete (UHPC) Matrix for Strength Enhancement and Shrinkage Reduction

**DOI:** 10.3390/ma18051135

**Published:** 2025-03-03

**Authors:** Zhijie Liu, Xibo Qi, Yuanhang Lv, Zhonghe Shui

**Affiliations:** 1 Sanya Science and Education Innovation Park, Wuhan University of Technology, Sanya 572004, China; 2 International School of Materials Science and Engineering, Wuhan University of Technology, Wuhan 430070, China; 3 State Key Laboratory of Silicate Materials for Architectures, Wuhan University of Technology, Wuhan 430070, China

**Keywords:** ultra-high-performance concrete, solid waste, volume stability, hydration kinetics, mechanical properties

## Abstract

Ultra-high-performance concrete is a high-strength and durable material widely used in infrastructure, but its high cement content raises environmental concerns, particularly in terms of CO₂ emissions and resource consumption. Phosphogypsum, an industrial by-product of phosphoric acid production, presents a sustainable alternative by partially replacing cement, thereby reducing cement demand and addressing solid waste disposal issues. This study investigates the effects of PG incorporation (0–40%) on hydration kinetics, mechanical properties, and volume stability in UHPC. The results indicate that increasing PG content delays hydration, affecting the induction period and peak hydration time. XRD and TG analysis confirm that PG modifies hydration product formation, influencing the development of key hydration phases. Strength tests reveal that moderate PG replacement (10–20%) maintains or improves long-term mechanical performance, while excessive PG replacement negatively impacts strength development. Additionally, PG effectively reduces autogenous shrinkage, improving the volume stability of UHPC. These findings highlight that PG can serve as a viable supplementary cementitious material in UHPC, contributing to both environmental sustainability and enhanced material performance.

## 1. Introduction

Ultra-high-performance concrete (UHPC) is an advanced cement-based composite that has attracted significant attention due to its excellent workability, mechanical properties, and durability [1,2,3,4,5]. Because of these exceptional properties, UHPC is particularly suitable for high-performance applications in the construction industry, such as high-rise buildings, bridge construction, and railway infrastructure. The development of UHPC is based on the theory of dense structure packing, which maximizes the packing density of individual materials, resulting in a dense, strong, and durable end product [6]. A key characteristic of UHPC is its very low water to binder ratio, typically below 0.2, which is critical to achieving its outstanding performance, particularly in terms of pressure resistance, impact resistance, and resistance to environmental influences, such as chloride penetration and freeze–thaw cycles. While UHPC offers exceptional properties, its production also has a significant impact on the environment. The concrete industry is one of the biggest sources of global CO_2_ emissions and accounts for around 6–7% of global emissions [7]. In addition, concrete production consumes large amounts of natural resources, particularly sand and stone, which are not renewable. In China alone, more than 9 billion tons of sand and stone are consumed annually, which contributes to environmental degradation [8]. These issues underscore the urgent need for sustainable alternatives in the construction industry.

One promising solution is to use solid industrial waste as a substitute for cementitious materials, such as ground granulated blast furnace slag, fly ash, silica dust, rice husk ash, coal bottom ash, steel production slag, and plastic waste [9,10]. By using these materials, it is possible to reduce the amount of cement required to produce UHPC, thereby alleviating the environmental impact associated with cement production and combating environmental pollution caused by the accumulation of solid waste. In addition, these industrial by-products improve the properties of concrete, such as durability, strength, and sustainability, thus contributing to more environmentally friendly building materials [11]. Solid industrial waste not only helps to reduce CO_2_ emissions but also reduces hydration heat in the early stages of cement hydration, optimizing the composition of hydration products and the microstructure of UHPC. This dual advantage improves both the environmental footprint of concrete production and the mechanical properties and durability of UHPC.

Phosphogypsum (PG) is an industrial by-product of the wet-process production of phosphoric acid in phosphorus chemical companies and is considered one of China’s most important industrial wastes [12]. The rapid expansion of the phosphate fertilizer industry has led to a sharp increase in PG emissions [13,14]. In China, the annual production of PG exceeds 55 million tons and the cumulative inventory exceeds 500 million tons, leading to serious environmental problems [15]. Hubei province, one of the most important production centers for phosphorus chemicals in China, has a particularly serious problem with PG inventories. In 2020, the province had accumulated 296 million tons of PG in 37 deposits covering an area of 15.8 square kilometers [16]. The presence of hundreds of chemical fertilizer companies in Hubei has also contributed to the large-scale production of PG as a by-product in the production of fertilizers, including sulfuric acid, ammonia, and phosphate fertilizers [17]. The untreated stacking of PG not only takes up large areas of land but also significantly affects the surrounding ecosystem as groundwater, soil, and air are polluted, leading to significant ecological and economic losses [18]. Because of these pressing environmental issues, researchers around the world are actively exploring strategies for using resources for PG [13]. Current applications focus primarily on the use of PG in the construction of plaster walls [19], in the production of panels [20], and for alkaline soil improvement [21]. However, these methods are still insufficient to significantly reduce PG stocks as their economic value is relatively low, which limits large-scale distribution [22,23]. Given the extent of PG accumulation and restrictions on existing methods of use, there is an urgent need to explore alternative high-quality applications that can efficiently consume PG while taking environmental concerns into account.

The application of PG in concrete is a sustainable approach to reducing environmental pollution while improving the recovery of solid industrial waste. Incorporating PG into concrete can minimize the consumption of natural resources, reduce production costs, and significantly reduce PG accumulation, improving resource efficiency. Over the years, numerous studies have investigated the potential of PG-based materials in construction, including their use as cement retarders [24,25], soil improvers [26], construction gypsum material [27], and road filler [28,29]. However, the overall utilization rate of PG is still below 40%, which underlines the need for further research in the area of high-quality applications. Since that cement and concrete are among the most commonly used building materials [30], the use of PG in these materials represents a viable solution for large-scale solid waste management.

One of the most intensively investigated approaches is the production of lightweight aggregates from PG. Wang et al. developed a lightweight aggregate that consists of over 80% PG [16]. It is characterized by low water absorption, high strength, and short curing time, and is, therefore, suitable for applications on road surfaces. Similarly, Zhang et al. produced an artificial aggregate with a composition of over 80% PG, supplemented by a small amount of slag and cement [31]. Their study revealed that the gypsum particles in the aggregate formed predominantly elongated structures, which took up the majority of the aggregate volume. Ding et al. also incorporated modified lightweight PG-based aggregates into the concrete, and the resulting material met the C30 strength grade for concrete after 28 days [32]. In addition, PG-based cold-bound aggregates with a PG content of 80% had good mechanical properties and high resistance to water erosion [13,32], which reinforced their potential for wider applications in concrete production.

In addition to its use in lightweight aggregates, PG was also investigated as a cementitious material in concrete production. However, some researchers have suggested that PG may not be suitable for direct use in concrete due to its potential effects on durability and stability [33,34,35]. Despite these concerns, other studies have shown that PG can be incorporated into cementitious systems through appropriate formulation and processing techniques. Wang et al. successfully developed PG-based cement by mixing 20% PG, 72% ground granulated blast furnace slag (GGBS), and 8% hard metal slag [36]. This cement formulation was used to produce C40 concrete, which proved its potential for higher strength applications. More recently, research has focused on the application of PG in UHPC [37,38,39,40,41,42]. Yang et al. found that although the mechanical strength of PG-containing UHPC was reduced at an early age, its long-term strength improved and its processability was significantly improved [40]. Their study also showed that UHPC still had excellent mechanical properties when cement replacement by PG was kept below 40%.

A review of the existing studies shows that extensive research has been conducted on the mechanical performance of UHPC with PG and the effect of curing conditions on PG-modified UHPC. However, the influence of high PG replacement levels on the volumetric stability of UHPC remains underexplored. Additionally, while previous studies have reported on the mechanical properties of UHPC containing PG, the fundamental mechanisms governing the relationship between PG content, hydration kinetics, and autogenous shrinkage are still not well understood. To address these gaps, this study systematically investigates the effects of high PG content (0–40% cement replacement) on the volumetric stability and mechanical properties of UHPC, with a particular focus on the hydration mechanisms that drive these changes. Under standard curing conditions, compressive strength tests were conducted to evaluate mechanical performance, while shrinkage tests were performed to assess volumetric stability. Furthermore, X-ray diffraction (XRD) and thermogravimetric (TG) analyses were employed to characterize the hydration products and their evolution over time. By clarifying the role of PG in UHPC, this research aims to provide a deeper understanding of its feasibility as a sustainable cementitious material and support its broader application in low-carbon construction.

## 2. Experimental Schemes

### 2.1. Raw Material

**Figure 1:**
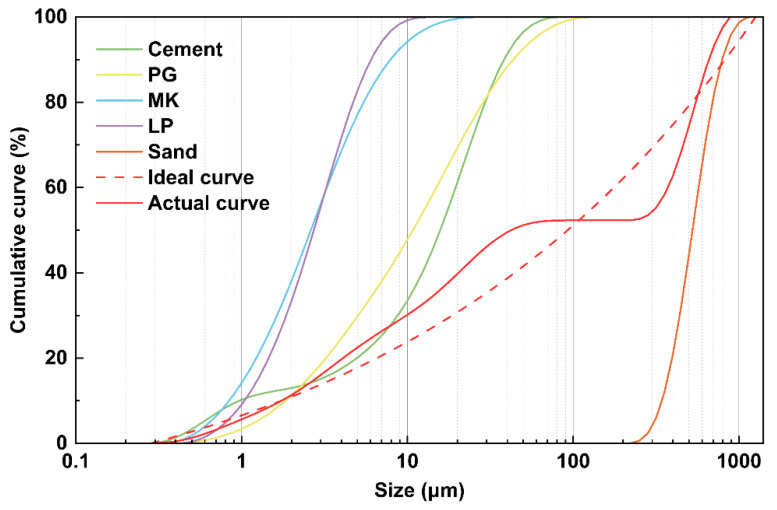
Particle size distributions of raw materials used in the study.

The raw materials used in this study include Huaxin P·II 52.5R ordinary Portland cement, Inner Mongolia Chaopai coal-series metakaolin (model 1300 kw), silica fume from Elkem (model 940U), limestone powder supplied by Xinmate, and a powdered polycarboxylate superplasticizer (BASF 325C). The phosphogypsum used in this study was supplied by Yichang Changyao Company. Table 1 shows the chemical properties of the binder materials analyzed by X-ray fluorescence (XRF), while Figure 1 illustrates the particle size distributions of the raw materials measured using a Malvern particle size analyzer with ethanol as the dispersion medium.

### 2.2. Mix Design and Specimen Preparation

To design the UHPC mixing ratios, the improved modified Anderson–Andersen model (MAA model) was applied [43,44]. Equation (1) was used as the objective function for particle packing, incorporating components, such as ordinary Portland cement, phosphogypsum, metakaolin, limestone powder, and sand. An optimization algorithm based on the least squares method, as expressed in Equation (2), was used to adjust the proportions of particles. This iterative process continued until the particle mixture’s accumulation curve achieved optimal alignment with the target curve. Equation (1) is as follows:

(1) $$P\left(D\right)=\frac{D^{q}-D_{min}^{q}}{D_{max}^{q}-D_{min}^{q}}$$

where *D* represents the particle size, with $D_{min}$ and $D_{max}$ denoting the minimum and maximum particle sizes, respectively. *P*(*D*) describes the proportion of the total particles that are smaller than a given particle size *D*. The distribution modulus q determines the ratio of fine to coarse particles in the mixture. When q>0.5, the mixture tends to be coarser, while lower values of q<0.25 lead to a mix richer in fine particles. Given the high proportion of fine particles typically used in UHPC production, a value of q=0.23 was selected based on previous studies. Equation (2) is as follows:

(2) $$\sum_{i=1}^{n}{\left[P_{mix}\left(D_{i}\right)-P_{tar}\left(D_{i}\right)\right]}^{2}\to min$$

where $P_{mix}$ represents the particle composition of the mixture, while $P_{tar}$ is the target distribution calculated using Equation (1). Using this method, the optimal mixing ratios for all tests were determined and are presented in Table 2. The superplasticizer (SP) accounted for 0.7% of the total weight of the cementitious material.

For mechanical and microstructural analysis, mortar samples were prepared for strength testing, while paste samples were used for component characterization, for example for XRD analysis. The mortar was poured into 40 mm × 40 mm × 160 mm molds, while the paste was formed into 40 mm cubes. After about 24 h, the samples were demolded and stored in a standard hardening room (20° C, 95% relative humidity) until they reached the required test age. To prepare the powder samples for analysis, the samples were taken from the hardening room at a certain age, with the outermost sample being removed 1 cm before processing. The remaining material was crushed, ground, and dipped in alcohol for 24 h to stop hydration. The samples were then vacuum dried for 48 h, sieved, and then subjected to further tests.

### 2.3. Compressive Strength

The compressive and flexural strength tests after 3, 7, and 28 days were carried out in accordance with the EN 196-1:2016 standard [45]. Each batch consisted of three 40 × 40 × 160 mm concrete prisms. For the three-point bending test, a vertical load of 50 N/s was applied over a span of 100 mm until the prism broke. The samples were then subjected to a compressive strength test with a load rate of 2.4 kN/s. The average compressive strength was determined by calculating the mean value of the results of all samples.

### 2.4. Setting Time

To evaluate the effect of PG on the setting time of UHPC, this study followed the EN 196-3:2016 standard [46]. Fresh mortar was poured into a mold (φ140 × 75 mm) and covered with a plastic film, maintaining a temperature of 20 °C. Measurements began at 2 h and were taken every 15 min until the penetration resistance reached 0.5 MPa, which was considered the setting time of UHPC.

### 2.5. Shrinkage Test

The contact shrinkage meter is used to measure the autogenous shrinkage of UHPC in accordance with the Chinese standard ASTM C1698-09 [47]. The bellows used for this test have an inner diameter of 22 mm, an outer diameter of 24 mm, and a length of 380 mm. After mixing, the fresh UHPC paste is poured into the bellows so that air bubbles can escape through the inner wall. Metal probes are attached to both ends of the bellows to measure the displacement in the first three days. The test is carried out in a controlled environment at a temperature of 20 ± 2 °C and a humidity of 60 ± 5%. The initial setting time is considered “time zero” for the autogenous shrinkage test.

### 2.6. Hydration Kinetics

The isothermal calorimetry tests were carried out using a TAM AIR isothermal calorimeter, keeping the sample temperature at a stable temperature of 25 °C. The heating rate was controlled between 0.1 °C/min and 2.0 °C/min with an accuracy of 0.1 °C.

### 2.7. TG–DTG

The TG-DSC tests were carried out using a STA449F3 synchronous thermal analyzer from Netzsch Instruments, Germany. The test was conducted in a nitrogen atmosphere, with a temperature range between 40 °C and 1000 °C and a heating rate of 10 °C/min.

### 2.8. X-Ray Diffraction

An X-ray diffraction analysis (XRD) was carried out to examine the mineral phases present in the samples. The XRD patterns were recorded using an Empyrean X-ray diffractometer with Cu Kα radiation, a scan range of 5° to 70° (2θ), and a sampling rate of 5°/min.

## 3. Results and Discussion

### 3.1. Heat of Hydration Analysis

**Figure 2:**
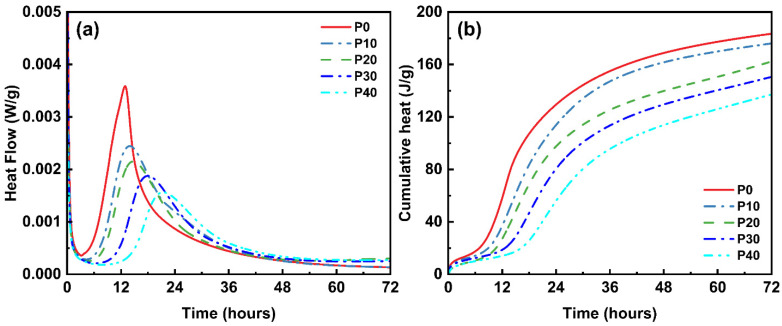
Isothermal calorimetry test results: (**a**) normalized heat flow; (**b**) cumulative heat.

Figure 2 shows the calorimetric test results, normalized per gram of binder. Data was collected over a 72 h hydration period to minimize external mixing effects. The heat flow curve has two distinct peaks. The larger peak corresponds to the dissolution and precipitation phase of the silicate, while the smaller peak corresponds to the formation of monosulfate. To evaluate the fluctuations in the hydration process of UHPC mixtures containing PG, several key parameters were calculated using the hydration curves, which are summarized in Table 3. The parameters t_A_ and t_B_ correspond to the end time of the induction period and the time at which the peak occurs, respectively. The heat flow values (dq/dt)_A_ and (dQ/dt)_B_ correspond to the heat flow rates at t_A_ and t_B_, respectively. Q_A_ and Q_B_ represent cumulative heat up to t_A_ and t_B_, respectively, while Q_T_ represents the total cumulative heat over the entire 72 h period.

Based on the data in Table 3, the hydration heat parameters of UHPC mixtures with different PG contents show significant trends that underline the influence of PG on the hydration process. As the PG content increases, both the induction period (t_A_) and the time until the hydration peak (t_B_) is reached are postponed to later times, which indicates a delayed start of hydration. In the control sample (P0), for example, the induction period is 3.20 h, and the peak value is 12.91 h. In contrast, at P40, the induction period is extended to 7.84 h, and the peak is observed at 21.37 h. This delay in hydration is related to the delaying effect of PG, which slows down the early stages of hydration due to the consumption of sulfate ions and the formation of AFt phases. By adding PG to cement, the hydration rate of tricalcium aluminate (C_3_A) is effectively regulated, preventing its rapid hydration, which, in turn, delays the cement setting time. This effect is evident in the setting time, which increases from 272 min in P0 to 386 min in P40. The additional sulfate ions from PG help consume C_4_AH_13_, which is considered one of the factors causing the instant setting of the concrete paste, thereby contributing to the controlled progression of hydration.

The heat flow peak, represented by (dq/dt)_B_, shows a similar trend. At P0, the heat flow at t_B_ is 3.5863 mW/g. It gradually decreases with increasing PG content and reaches a low of 1.5633 mW/g at P40. This reduction indicates that the total exothermic activity of the hydration process decreases as the PG content increases. This can be attributed to the lower availability of reactive sulfate ions and the delayed formation of C-S-H phases. Cumulative heat (Q_A_, Q_B_, and Q_T_) further supports this trend, with the total cumulative heat released by P0 after 72 h being 183.5 J/g, while for P40 it decreases to 137.2 J/g.

This pattern suggests that higher PG replacement values result in lower heat output during the hydration process, which is likely due to slower reaction kinetics and the dilution effect caused by the additional gypsum and reduced cement content. However, despite the lower total heat release, the hydration process with PG results in more stable and controlled heat generation with a more gradual increase and a lower peak heat flow. This effect can be attributed to the continued formation of AFt phases and the slower dissolution of the silicate phases, which can help to mitigate shrinkage during the early aging process and to regulate heat output, thereby improving the volume stability of the UHPC product. In summary, although a higher PG content delays the hydration process and reduces the total heat released, it also results in more controlled and stable hydration development. This dual function of PG—it alleviates both the heat release and the shrinkage of hydration products—makes it a valuable material for improving the volume stability of UHPC.

### 3.2. XRD Analysis

**Figure 3:**
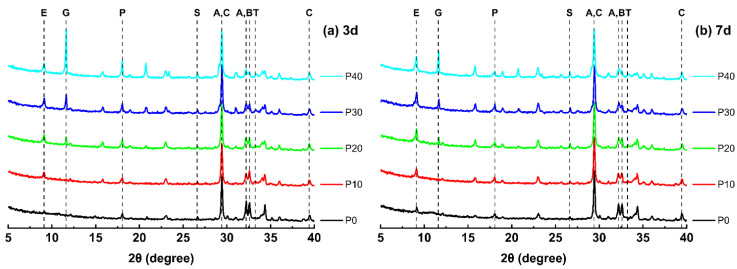
XRD patterns of UHPC at 3 and 7 days with varying PG replacement levels. Note: (A: C_3_S,PDF#86-0402; B: C_2_S,PDF#83-0461; C: calcite,PDF#83-0577; S: quartz,PDF#46-1045; T: C_3_A,PDF#33-0251; E: ettringite. (AFt),PDF#41-1451; P: Portland (CH),PDF#76-0571; G: gypsum,PDF#33-0311).

Figure 3 shows the XRD patterns of UHPC samples after 3 and 7 days and shows the phase evolution with different PG replacement values. After 3 days, the gypsum peak in P10 is significantly lower, which indicates that most of the plaster has been used. However, at P20 and higher replacement levels, the gypsum peak gradually increases, which indicates that an excessive addition of PG exceeds the consumption capacity. After 7 days, plaster peaks have continued to fall compared to after 3 days, and even at P20, the peak value is significantly lower, which confirms continuous gypsum consumption over time.

In addition, the portlandite peak (CH) rises after 3 days with higher PG exchange values, with P40 having the highest CH peak intensity. This is because C_3_A not only reacts to calcium aluminate hydrate (C_4_AH_13_) in the presence of gypsum, but also reacts with gypsum, producing additional CH. The relevant responses are as follows:

$$C_{3}A+CH+12H\to C_{4}AH_{13}$$

$$C_{4}AH_{13}+3C\bar{S}H_{2}+14H\to C_{3}A\cdot 3C\bar{S}\cdot H_{32}+CH$$

These reactions contribute to the continuous formation of CH in the early stages of hydration. This suggests that the presence of PG influences CH formation at an early age. However, after 7 days, the peak CH intensity decreases in all samples, which indicates continued pozzolanic reactions or further hydration processes that consume CH. The active silica in metakaolin reacts with CH in a pozzolanic reaction to form additional C-S-H, which can contribute to strength development. In addition, the active aluminum in the system can react with CH, contributing to the formation of C_3_A and potentially other hydration products, such as C-A-S-H gel and AFm phases. These reactions are typically presented as follows:

$$SiO_{2}+CH+H_{2}O\to C\text{-}S\text{-}H\;(\mathrm{calcium}\;\mathrm{silicate}\;\mathrm{hydrate})$$

$$Al_{2}O_{3}+3Ca{\left(OH\right)}_{2}\to 3CaO\cdot Al_{2}O_{3}+3H_{2}O$$

These processes result in the formation of additional C-S-H phases, which increase the strength and durability of the UHPC. These results underscore the dynamic phase transformations in UHPC during PG change and suggest that an optimal replacement level is required to balance gypsum consumption and the formation of hydration products.

### 3.3. Thermal Analysis

**Figure 4:**
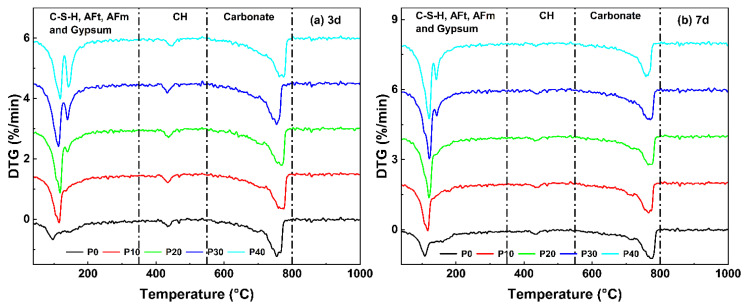
DTG curves of UHPC mixed with PG at 3 and 7 days.

In order to further investigate the hydration process of UHPC with different PG contents, the development of the hydrated phases was investigated using thermal analysis, which was conducted in the temperature range of 40–1000 °C. The experimental results are shown in Figure 4. It was observed that all samples have significant temperature peaks in the temperature range of 50 to 200 °C, primarily due to dehydration and physical water loss of ettringite (AFt) and C-S-H gels. This suggests the presence of early hydration products in the UHPC matrix. In particular, for samples with higher PG contents (P20–P40), an additional side peak at around 140 °C was observed after 3 days, which corresponds to the decomposition of residual gypsum. This observation is consistent with the XRD results and confirms that the excess PG in these samples was not completely consumed during the early hydration phase. A significant degradation peak was found between 400–500 °C, which corresponds to the endothermic degradation of CH. This also confirms the XRD results, where higher CH peaks were observed in the early stages of hydration, particularly in samples with higher PG content. In addition, there is a broad peak between 700–800 °C, which is related to the thermal decomposition of CaCO_3_ (calcium carbonate), which was likely caused by the carbonization of CH over time. These results confirm that PG influences the hydration process by altering the formation and consumption of hydration products and affecting the thermal behavior of UHPC over time.

**Figure 5:**
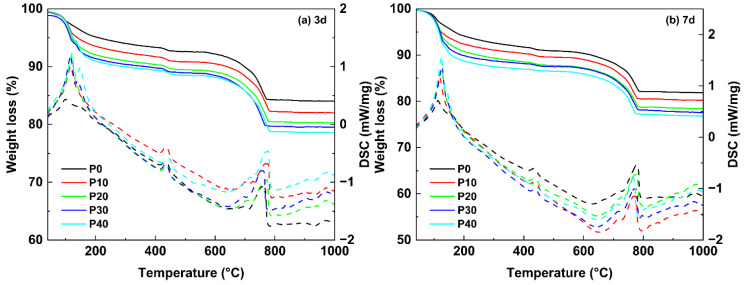
TG and DSC curves of UHPC mixed with PG at 3 and 7 days. Weight loss: solid line; DSC: dashed line.

**Figure 6:**
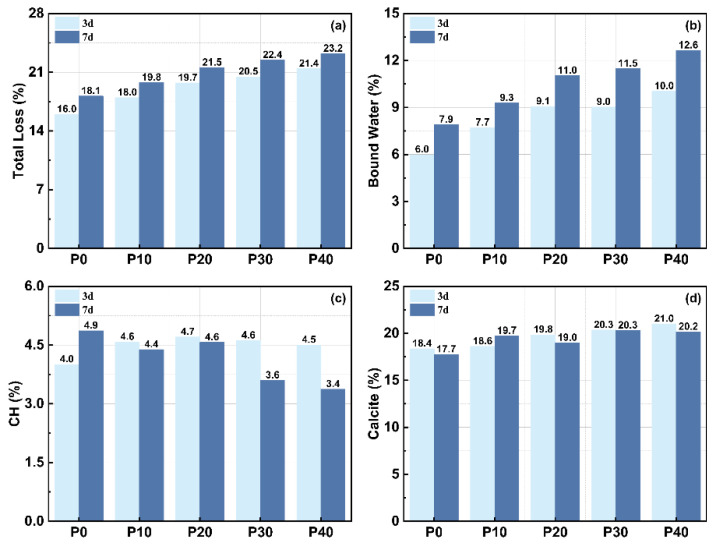
The variation in total weight loss and chemical components calculated from TGA and calcination results. (**a**) Total loss, (**b**) Bound water, (**c**) Calcium hydroxide content, (**d**) Calcite content.

Figure 5 shows the TG and DSC curves of UHPC mixed with PG after 3 and 28 days. Figure 6 shows the variation in total weight loss and chemical components calculated using TGA and calcination results. The specific calculation formula can be found in reference [43]. These results illustrate the impact of PG on the hydration process and the evolution of phase composition over time.

The total weight loss increases with the replacement of PG, which indicates an increased formation of hydration products. After 3 days, total weight loss increases from 15.98% (P0) to 21.40% (P40), while after 7 days it continues to increase from 18.15% (P0) to 23.21% (P40). A similar trend is observed in the content of bound water, which stands for chemically bound water in hydration products, such as C-S-H and AFt. After 3 days, the bound water content rises from 5.99% (P0) to 10.04% (P40), and after 7 days it rises from 7.90% (P0) to 12.64% (P40). These results suggest that as PG content increases and hardening age increases, more free water participates in hydration reactions to form bound water. The increase in bound water content also confirms that PG promotes the formation of hydration products, particularly AFt, which requires additional water for its crystalline structure. As a result, higher hydration activity results in higher mass loss during thermal decomposition.

The CH content shows a clear trend in various stages of hydration. After 3 days, the CH content with PG initially rises and reaches a peak of 4.71% (P20) before falling slightly to 4.50% (P40). This suggests that the reaction involving PG initially contributes to CH formation. However, as hydration progresses, CH is consumed in secondary reactions involving PG and metakaolin, resulting in a decrease in CH content. After 7 days, a similar trend can be observed: CH peaks at 4.86% (P0) but drops to 3.37% (P40) as more PG is replaced. This suggests that different levels of PG influence the hydration process at various stages and change the internal proportions of hydration products over time.

The calcite content remains relatively stable in all samples, as most calcite comes from raw materials, and carbonization during sample preparation only contributes slightly to this. After 3 days, the calcite content fluctuates between 18.36% (P0) and 20.99% (P40), while after 7 days it is between 17.73% (P0) and 20.15% (P40). The slight fluctuations can be attributed to differences in the availability of CH for carbonization, with higher levels of PG potentially influencing the carbonization rate. Overall, however, the results suggest that the carbonation effects are not significantly affected by the replacement of PG.

### 3.4. Mechanical Properties

**Figure 7:**
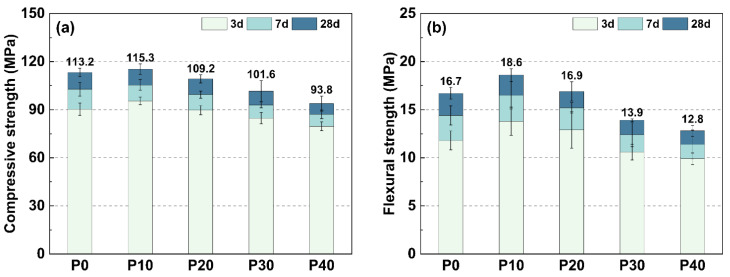
Compressive and flexural strength development of UHPC with varying PG replacement levels. (**a**) Compressive strength; (**b**) Flexural strength.

Figure 7a shows the compressive strength of UHPC samples with different PG replacement values (P0 to P40), measured after 3, 7, and 28 days. As the PG content increased, a general decline in compressive strength was observed at all ages. After 3 days, the strength dropped from 90.3 MPa for P0 to 79.6 MPa for P40. A similar trend was observed after 7 days, where the increase in strength declined from 3 to 7 days as the PG content increased. In particular, P0 showed an increase of 12.4 MPa, while P40 only had an increase of 7.5 MPa. After 28 days, this trend continued, with P0 increasing by a further 10.5 MPa, whereas P40 only increased by 6.7 MPa. The total compressive strength after 28 days shows that P10 achieved the highest strength at 115.3 MPa and, thus, slightly exceeded the limit value (P0) at 113.2 MPa, while P20, P30, and P40 each reached 109.2 MPa, 101.6 MPa, and 93.8 MPa, respectively.

Figure 7b shows the bending strength of UHPC samples after 3, 7, and 28 days. The bending strength after 3 days rose from 11.8 MPa for P0 to a peak of 13.8 MPa for P10, followed by a gradual decline with increasing PG content, with P40 reaching 9.9 MPa. The increase in strength from 3 to 7 days also decreased as the PG content increased. P0 increased by 2.6 MPa and P10 had the highest increase at 2.7 MPa, while P40 had the lowest value at 1.5 MPa. After 28 days, the bending strength continued to increase, but at a reduced rate, with P0 increasing by 2.3 MPa and P40 only by 1.4 MPa. The total bending strength after 28 days was highest for P10 at 18.6 MPa, followed by P0 (16.7 MPa), P20 (16.9 MPa), P30 (13.9 MPa), and P40 (12.8 MPa).

These results suggest that the replacement of PG influences both the development of compressive and flexural strength in UHPC. While higher PG content has a negative effect on strength development at an early age due to its retarding effect on cement hydration, moderate replacement amounts (such as P10) can improve long-term mechanical properties. This improvement is likely due to secondary hydration reactions and microstructure refinement, which contribute to strength development over time. However, excessive addition of PG results in a decrease in both compressive and flexural strength, suggesting that there is an optimal substitute grade to balance strength performance and continued use of PG at UHPC. Overall, these findings underscore the existence of an optimal PG replacement level (approximately P10), which effectively balances the sustainable utilization of PG with the mechanical performance of UHPC.

### 3.5. Autogenous Shrinkage

**Figure 8:**
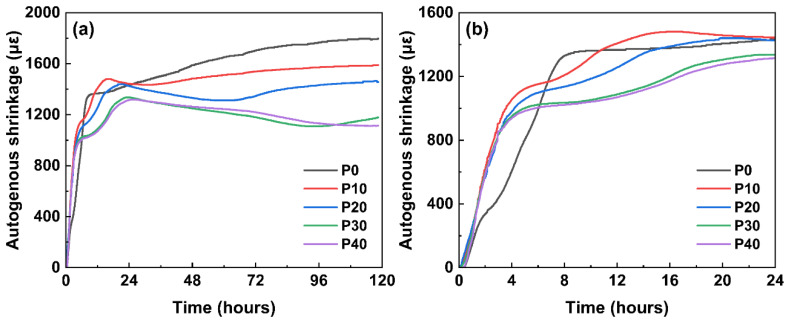
Autogenous shrinkage of mixtures with PG: (**a**) overall diagram; (**b**) partial enlarged image.

Autogenous shrinkage measurements were carried out to assess the volume stability of UHPC with PG. The results are shown in Figure 8. It is obvious that the autogenous shrinkage of all UHPC samples increases over time. In addition, the results confirm that replacing cement with PG significantly reduces the autogenous shrinkage of UHPC. In particular, replacing 10–40% of cement with PG reduces the 5-day shrinkage rate by 11.6–38.0%, which demonstrates the effectiveness of PG in reducing shrinkage at an early age. Based on the shrinkage curves, it can be seen that UHPC mixtures containing PG show slight elongation after a certain degree of hydration. This expansion can be attributed to the continuous formation of AFt, which is due to the excess sulfate ions present in the system. In addition, the presence of limestone powder helps stabilize AFt by reacting faster with hydrated calcium aluminate phases than converting AFt to AFm. This process hinders the conversion of AFt to AFm and, thus, increases the stability of ettringite. As a result, the hydration process is moderated, and the cement dilution effect further contributes to improving the volume stability of UHPC. Based on the experimental results, a high PG content is effective to mitigate autogenous shrinkage and improve the volume stability and durability of UHPC, ensuring its long-term structural performance.

## 4. Conclusions

In this study, the effect of PG as a cement substitute in UHPC was investigated, focusing on its influence on hydration kinetics, mechanical properties, and volume stability. The most important results can be summarized as follows:

1. Hydration process: The results of isothermal calorimetry showed that an increasing PG content delays hydration, which is reflected in the extended induction period and the hydration peak time. This delay is attributed to the sulfate regulation of C_3_A hydration, which prevents the cement from setting rapidly and improves volume stability.
2. Phase evolution: XRD and TG results confirmed that, under room temperature conditions, PG alters the formation of hydration products, with the formation of AFt increasing at early stages and secondary hydration reactions consuming CH over time, resulting in microstructure refinement.
3. Mechanical properties: The compressive and flexural strength tests showed that although strength development at an early age was hindered by the retarding effect of PG, mechanical performance remained comparable or even better in the long term with moderate replacement quantities. The results suggest that a PG substitute (≤10–20%) optimizes both processability and long-term strength, whereas excessive replacement results in a decrease in strength.
4. Autogenous shrinkage and volume stability: PG significantly reduces shrinkage at an early age, with a PG replacement of 10 to 40% reducing shrinkage by 11.6–38.0% within 5 days. This is attributed to the micro-expansion effect of PG in early hydration phases. In addition, the limestone powder in the system prevents the conversion of AFt to AFm, further improving shrinkage resistance and volume stability.

## Figures and Tables

**Table 1:** Chemical properties of the binder materials used in this study (wt.%).

Materials	CaO	SiO_2_	Al_2_O_3_	Fe_2_O_3_	Na_2_O	MgO	SO_3_	TiO_2_	L.O.I.
Cement	61.91	20.17	5.09	3.68	0.16	0.85	2.41	0.29	5.46
PG	36.07	5.34	0.83	0.41	0.30	0.77	32.05	0.11	24.12
MK	0.23	49.43	46.57	0.76	0.18	0.15	0.04	1.23	1.42
LP	56.55	0.49	0.07	-	-	0.29	-	-	42.6

**Table 2:** The mix proportion of UHPC matrix (kg/m^3^).

Group	Cement	PG	LP	MK	Sand	w/b	SP
P0	1015	0	271	258	1315	0.2	10.8
P10	914	101	271	258	1315	0.2	10.8
P20	812	203	271	258	1315	0.2	10.8
P30	711	304	271	258	1315	0.2	10.8
P40	609	406	271	258	1315	0.2	10.8

**Table 3:** Parameters calculated from the hydration curves of mixtures with PG.

	t_A_	(dQ/dt)_A_	Q_A_	t_B_	(dQ/dt)_B_	Q_B_	Q_T_
	(h)	(mW/g)	(J/g)	(h)	(mW/g)	(J/g)	(J/g)
P0	3.20	0.3570	12.9	12.91	3.5863	69.0	183.5
P10	4.27	0.2840	12.4	13.96	2.4457	52.7	176.1
P20	4.67	0.2524	9.4	14.72	2.1529	45.9	162.3
P30	6.27	0.2118	12.7	17.98	1.8768	46.6	150.6
P40	7.84	0.1842	11.2	21.37	1.5633	42.4	137.2

## Data Availability

The data presented in this study are available on request from the corresponding author.

## References

[1] Sun T., Wang X., Maimaitituersun N., Dong S., Li L., Han B. (2024). Synergistic effects of steel fibers and steel wires on uniaxial tensile mechanical and self-sensing properties of UHPC. Constr. Build. Mater..

[2] Mo Z., Gao X., Su A. (2020). Mechanical performances and microstructures of metakaolin contained UHPC matrix under steam curing conditions. Constr. Build. Mater..

[3] Kim S., Yoo D.-Y., Kim M.-J., Banthia N. (2019). Self-healing capability of ultra-high-performance fiber-reinforced concrete after exposure to cryogenic temperature. Cem. Concr. Compos..

[4] Zhuang W., Li S., Wang Z., Zhang Z., Yu Q. (2022). Impact of micromechanics on dynamic compressive behavior of ultra-high performance concrete containing limestone powder. Compos. Part B-Eng..

[5] Wang J., Dong S., Pang S.D., Yu X., Han B., Ou J. (2022). Tailoring Anti-Impact Properties of Ultra-High Performance Concrete by Incorporating Functionalized Carbon Nanotubes. Engineering.

[6] Lu J.-X., Shen P., Sun Y., Poon C.S. (2022). Strategy for preventing explosive spalling and enhancing material efficiency of lightweight ultra high-performance concrete. Cem. Concr. Res..

[7] Mo K.H., Johnson Alengaram U., Jumaat M.Z., Yap S.P. (2015). Feasibility study of high volume slag as cement replacement for sustainable structural lightweight oil palm shell concrete. J. Clean. Prod..

[8] Jin Z., Ma B., Su Y., Lu W., Qi H., Hu P. (2020). Effect of calcium sulphoaluminate cement on mechanical strength and waterproof properties of beta-hemihydrate phosphogypsum. Constr. Build. Mater..

[9] Raheem A.A., Abdulwahab R., Kareem M.A. (2021). Incorporation of metakaolin and nanosilica in blended cement mortar and concrete—A review. J. Cleaner Prod..

[10] Saidin S.S., Kudus S.A., Jamadin A., Anuar M.A., Amin N.M., Ibrahim Z., Zakaria A.B., Sugiura K. (2022). Operational modal analysis and finite element model updating of ultra-high-performance concrete bridge based on ambient vibration test. Case Stud. Constr. Mater..

[11] Guo M., Hu B., Xing F., Zhou X., Sun M., Sui L., Zhou Y. (2020). Characterization of the mechanical properties of eco-friendly concrete made with untreated sea sand and seawater based on statistical analysis. Constr. Build. Mater..

[12] Zhou S., Li X., Zhou Y., Min C., Shi Y. (2020). Effect of phosphorus on the properties of phosphogypsum-based cemented backfill. J. Hazard. Mater..

[13] Liu Y., Zhang D., You L., Luo H., Xu W. (2022). Recycling phosphogypsum in subbase of pavement: Treatment, testing, and application. Constr. Build. Mater..

[14] Wei Z., Deng Z. (2022). Research hotspots and trends of comprehensive utilization of phosphogypsum: Bibliometric analysis. J. Environ. Radioact..

[15] Chuan L.M., Zheng H.G., Zhao J.J., Wang A.L., Sun S.F. (2018). Phosphogypsum production and utilization in China. IOP Conf. Ser. Mater. Sci. Eng..

[16] Wang C., Chen S., Huang D., Huang Q., Li X., Shui Z. (2023). Safe environmentally friendly reuse of red mud modified phosphogypsum composite cementitious material. Constr. Build. Mater..

[17] Xie Y., Sun T., Shui Z., Ding C., Li W. (2022). The impact of carbonation at different CO_2_ concentrations on the microstructure of phosphogypsum-based supersulfated cement paste. Constr. Build. Mater..

[18] Wang Z., Shui Z., Sun T., Li X., Zhang M. (2022). Recycling utilization of phosphogypsum in eco excess-sulphate cement: Synergistic effects of metakaolin and slag additives on hydration, strength and microstructure. J. Clean. Prod..

[19] Contreras Llanes M., Pérez López R., Gázquez González M.J., Morales Flórez V., Santos A., Esquivias L., Bolívar Raya J.P. (2015). Fractionation and fluxes of metals and radionuclides during the recycling process of phosphogypsum wastes applied to mineral CO_2_ sequestration. Waste Manag..

[20] Neto J.S.A., Bersch J.D., Silva T.S., Rodríguez E.D., Suzuki S., Kirchheim A.P. (2021). Influence of phosphogypsum purification with lime on the properties of cementitious matrices with and without plasticizer. Constr. Build. Mater..

[21] Wang J., Tan H., He X., Zhang J., Jian S., Du C., Deng X. (2022). Influence of wet grinded slag on the hydration of phosphogypsum-slag based cement and its application in backfill tailings. Constr. Build. Mater..

[22] Huang X., Li J., Jiang W., Chen Z., Wan Y., Xue Q., Liu L., Poon C.S. (2022). Recycling of phosphogypsum and red mud in low carbon and green cementitious materials for vertical barrier. Sci. Total Environ..

[23] Cao J., Wang Z., Ma X., Yang X., Zhang X., Pan H., Wu J., Xu M., Lin L., Zhang Y. (2022). Promoting coordinative development of phosphogypsum resources reuse through a novel integrated approach: A case study from China. J. Cleaner Prod..

[24] Akın Altun İ., Sert Y. (2004). Utilization of weathered phosphogypsum as set retarder in portland cement. Cem. Concr. Res..

[25] Kacimi L., Simon-Masseron A., Ghomari A., Derriche Z. (2006). Reduction of clinkerization temperature by using phosphogypsum. J. Hazard. Mater..

[26] Enamorado S., Abril J.M., Delgado A., Más J.L., Polvillo O., Quintero J.M. (2014). Implications for food safety of the uptake by tomato of 25 trace-elements from a phosphogypsum amended soil from SW spain. J. Hazard. Mater..

[27] Wei Z., Zhang Q., Li X. (2021). Crystallization kinetics of α-hemihydrate gypsum prepared by hydrothermal method in atmospheric salt solution medium. Crystals.

[28] Diouri C., Echehbani I., Lahlou K., Omari K.E., Alaoui A. (2022). Valorization of moroccan phosphogypsum in road engineering: Parametric study. Mater. Today Proc..

[29] Meskini S., Samdi A., Ejjaouani H., Remmal T. (2021). Valorization of phosphogypsum as a road material: Stabilizing effect of fly ash and lime additives on strength and durability. J. Cleaner Prod..

[30] Aslam M., Shafigh P., Jumaat M.Z., Lachemi M. (2016). Benefits of using blended waste coarse lightweight aggregates in structural lightweight aggregate concrete. J. Cleaner Prod..

[31] Zhang L., Mo K.H., Yap S.P., Gencel O., Ling T.-C. (2022). Mechanical strength, water resistance and drying shrinkage of lightweight hemihydrate phosphogypsum-cement composite with ground granulated blast furnace slag and recycled waste glass. Constr. Build. Mater..

[32] Ding C., Sun T., Shui Z., Xie Y., Ye Z. (2022). Physical properties, strength, and impurities stability of phosphogypsum-based cold-bonded aggregates. Constr. Build. Mater..

[33] Pinto S.R., Angulski da Luz C., Munhoz G.S., Medeiros-Junior R.A. (2020). Durability of phosphogypsum-based supersulfated cement mortar against external attack by sodium and magnesium sulfate. Cem. Concr. Res..

[34] Li H., Xu F., Li B., Sun T., Huang X., Zhu J., Peng C., Lin J., Chen Z. (2022). Investigation on mechanical properties of excess-sulfate phosphogypsum slag cement: From experiments to molecular dynamics simulation. Constr. Build. Mater..

[35] Chen J., Jiang M. (2009). Long-term evolution of delayed ettringite and gypsum in portland cement mortars under sulfate erosion. Constr. Build. Mater..

[36] Wang Y., Xu L., He X., Su Y., Miao W., Strnadel B., Huang X. (2022). Hydration and rheology of activated ultra-fine ground granulated blast furnace slag with carbide slag and anhydrous phosphogypsum. Cem. Concr. Compos..

[37] Peng Y., Li X., Liu Y., Zhan B., Xu G. (2020). Optimization for mix proportion of reactive powder concrete containing phosphorous slag by using packing model. J. Adv. Concr. Technol..

[38] Peng Y., Zhang J., Liu J., Ke J., Wang F. (2015). Properties and microstructure of reactive powder concrete having a high content of phosphorous slag powder and silica fume. Constr. Build. Mater..

[39] Huang Y., Chen G., Yang R., Yu R., Xiao R., Wang Z., Xie G., Cheng J. (2023). Hydration kinetics and microstructure development of ultra-high performance concrete (UHPC) by high volume of phosphorus slag powder. Cem. Concr. Compos..

[40] Rui Y., Rui Y., Zhonghe S., Xu G., Xunguang X., Xiangbin Z., Yunyao W., Yongjia H. (2019). Low carbon design of an Ultra-High Performance Concrete (UHPC) incorporating phosphorous slag. J. Cleaner Prod..

[41] Chen G., Huang Y., Yang R., Yu R., Xiao R., Wang Z., Ke X., Xie G., Cheng J., Bao M. (2023). Comparative study on mechanical properties and microstructure development of ultra-high performance concrete incorporating phosphorous slag under different curing regimes. Constr. Build. Mater..

[42] Shi Y., Matsui I., Feng N. (2002). Effect of compound mineral powders on workability and rheological property of HPC. Cem. Concr. Res..

[43] Liu Z., Qi X., Shui Z., Lv Y. (2024). The preparation, property and hydration mechanism of ultra high performance concrete with metakaolin substitution for silica fume. J. Build. Eng..

[44] Liu Z., Qi X., Yu Z., Gao X., Shui Z. (2024). Development and properties of cost-effective self-sensing Ultra-High Performance Fiber-Reinforced Concrete (UHPFRC) incorporating steel slags. Constr. Build. Mater..

[45] (2016). Methods of Testing Cement—Part 1: Determination of Strength.

[46] (2016). Methods of Testing Cement—Part 3: Determination of Setting Times and Soundness.

[47] (2014). Standard Test Method for Autogenous Strain of Cement Paste and Mortar.

